# Hepatitis E virus and Klebsiella pneumoniae co-infection detected by metagenomics next-generation sequencing in a patient with central nervous system and bloodstream Infection: a case report

**DOI:** 10.1186/s12879-023-08850-4

**Published:** 2024-01-02

**Authors:** Manman Cui, Wei Sun, Yuan Xue, Jiangnan Yang, Tianmin Xu

**Affiliations:** 1 Department of Infectious Diseases, the Third People’s Hospital of Changzhou, Changzhou, China; 2 Department of Science and Education, the Third People’s Hospital of Changzhou, Changzhou, China; 3 Department of Medicine, Dinfectome Inc, Nanjing, China

**Keywords:** Hepatitis E virus, *Klebsiella pneumoniae*, Meningitis, Case report, Metagenomic next-generation sequencing

## Abstract

**Background:**

Hepatitis E virus (HEV) is the most common cause of acute viral hepatitis worldwide with major prevalence in the developing countries and can cause extrahepatic disease including the nervous system. Central nervous system infections caused by HEV are rare and caused by HEV together with other bacteria are even rarer.

**Case presentation:**

A 68-year-old man was admitted to the hospital due to a headache lasting for 6 days and a fever for 3 days. Lab tests showed significantly raised indicators of inflammation, cloudy cerebrospinal fluid, and liver dysfunction. Hepatitis E virus and *Klebsiella pneumoniae* were identified in the blood and cerebrospinal fluid using metagenomic next-generation sequencing. The patient received meropenem injection to treat *K. pneumoniae* infection, isoglycoside magnesium oxalate injection and polyene phosphatidylcholine injection for liver protection. After ten days of treatment, the patient improved and was discharged from the hospital.

**Conclusion:**

Metagenomic next-generation sequencing, which can detect various types of microorganisms, is powerful for identifying complicated infections.

## Background

The Hepatitis E virus (HEV) stands as the leading cause of acute viral hepatitis worldwide, notably in developing nations [1, 2]. According to the World Health Organization (WHO), every year there are an estimated 20 million HEV infections worldwide, leading to an estimated 3.3 million symptomatic cases of hepatitis E (https://www.who.int/en/news-room/fact-sheets/detail/hepatitis-e). Most cases of acute HEV infection resolve spontaneously and do not require treatment, life-threatening acute liver failure may occur in some cases, especially pregnant women and immunocompromised patients.

HEV infection not only triggers hepatitis but also associates with neurological issues like Guillain − Barré syndrome (GBS), neuralgic amyotrophy, and encephalitis/myelitis [3]. However, the exact mechanisms behind these neurological symptoms remain unclear. Earlier case reports have shown that HEV can be found in the cerebrospinal fluid (CSF) of patients displaying neurological symptoms [4–7]. Laboratory investigations revealed that HEV has the capacity to harm tight junction proteins like Claudin5, Occludin, and ZO-1 (zonula occludens-1), disrupting the blood–brain barrier and allowing entry into the cerebrospinal fluid. Additionally, HEV can replicate and proliferate within nerve cells, resulting in central nervous system infections [8–10].

*Klebsiella pneumoniae*, a Gram-negative bacterium, frequently exists in the environment. Humans, being the primary host, commonly harbor *K. pneumoniae* in the nasopharynx and gastrointestinal tract. In healthcare facilities, the risk of colonization and consequent infection is elevated, potentially leading to hospital-based outbreaks [11]. Individuals with compromised immune systems are particularly susceptible to various infections, with respiratory, urinary tract, and bloodstream infections being most prevalent [12]. In the context of central nervous system infections, *K. pneumoniae* is the primary causative agent of bacterial meningitis. Among Chinese patients diagnosed with bacterial meningitis, *K. pneumoniae* prevalence reached a notable 11.3% [13]. The substantial occurrence and fatality rate of *K. pneumoniae*-induced meningitis warrant serious attention.

To date, instances of concurrent HEV and *K. pneumoniae* infections have not been reported. Therefore, we present a case involving a combined infection affecting the central nervous system and bloodstream caused by both HEV and *K. pneumoniae*, which was successfully treated. In this case, CSF and blood samples underwent analysis through mNGS. This approach enabled the swift identification of pathogens, thus facilitating early treatment and improving the patient's prognosis.

## Case presentation

**Fig. 1:**
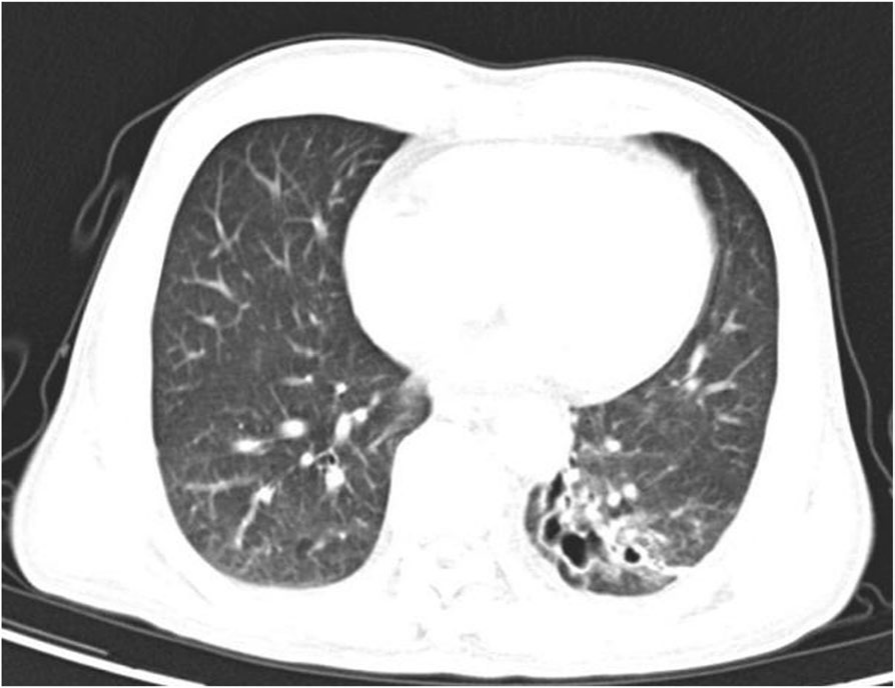
Chest CT

A 68-year-old man suddenly experienced fever and headache, with a slightly stiff neck. He had a history of bronchiectasis for over 40 years and had frequently used ciprofloxacin and amoxicillin to treat bronchiectasis-related infections. A chest CT scan displayed bronchiectasis in the lower left lobe (Fig. 1).

On April 5, 2023, he underwent testing at the hospital. Biochemical assessments indicated heightened alanine aminotransferase (ALT: 1780 U/L), signifying severe liver damage. Magnetic resonance imaging (MRI) showcased age-related brain changes and bilateral sphenoid sinusitis. On April 7, he was transferred to the Third People’s Hospital of Changzhou. Following admission, he exhibited a peak body temperature of 40 °C, noticeable headaches, and had four instances of watery yellow diarrhea. Inflammatory markers were significantly elevated (WBC 10.03E + 09/L, CRP 100.69 mg/L, PCT 2.64 ng/ml, IL-6 114.9 pg/mL). By April 8, the cerebrospinal fluid (CSF) had a light-yellow appearance, and CSF testing revealed increased WBC count (2.126E + 09/L), predominant multinucleated cells (86.3%), heightened protein (2.86 g/L), and decreased glucose (2.00 mmol/L) and chloride (113.3 mmol/L). Further test outcomes are presented in Table 1. Based on these findings, the diagnosis pointed to a central nervous system infection, with a strong likelihood of suppurative meningitis. The patient also exhibited irregular liver function and notably elevated liver enzymes. While tests for hepatitis A, B, C, and D antibodies returned negative, the hepatitis E IgM level was 8.086 S/co, suggesting a potential HEV infection.

**Table 1:** Laboratory indicators on the first day of admission

**CSF test**	
Pressure	150mmH_2_O
Paneth's test	weak positive
WBC	2.126E + 09/L
Mononuclear cell	13.70%
Protein	2.86 g/L
Glucose	2.00 mmol/L
Cl	113.3 mmol/L
cryptococcal antigen	Negative
Xpert MTB	Negative
**Blood routine**	
WBC	10.03E + 09/L
Neutrophil	83.00%
Lymphocyte	8.30%
Eosinophil	0.10%
RBC	4.22E + 12/L
Hemoglobin	125.0 g/L
Platelets	95E + 09/L
B-type natriuretic peptide	143.7 pg/mL
CD3%	40.18%
CD3 +	564/μL
CD4%	16.61%
CD4 +	233/μL
CD8%	23.82%
CD8 +	334/μL
CD19%	5.69%
CD19 +	80/μL
Biochemistry	
Globulin	23.3 g/L
Glucose	6.30 mmol/L
CRP	100.69 mg/L
Amyloid A	378.70 mg/L
Uric acid	162.3μmol/l
Ca	1.95 mmol/L
Iron	5.14μmol/L
D-dimer	2.27μg/ml
PCT	2.64 ng/ml
IL-6	114.9 pg/mL
**Coagulation system**	
PT	13.50 S
PT-INR	1
**Virological examination**	
HBc Ab	Negative
HAV Ab	Negative
HCV Ab	Negative
HDV Ab	Negative
HGV Ab	Negative
HIV Ab	Negative
HEV IgM	7.750 S/co
HEV IgG	Negative
**Liver function**	
Alanine aminotransferase (ALT)	423.9 U/L
Aspartate aminotransferase (AST)	53 U/L
Alkaline phosphatase	235 U/L
Gamma-glutamyl transferase (GGT)	241.2 U/L
Total bilirubin	17.7 μmol/L
Direct bilirubin	11.6 μmol/L
Total protein	52.0 g/L
Albumin	28.7 g/L
Prealbumin	5.4 mg/dL
Glycocholic acid	18.4 mg/L
Total bile acid	27.3 μmol/L

**Fig. 2:**
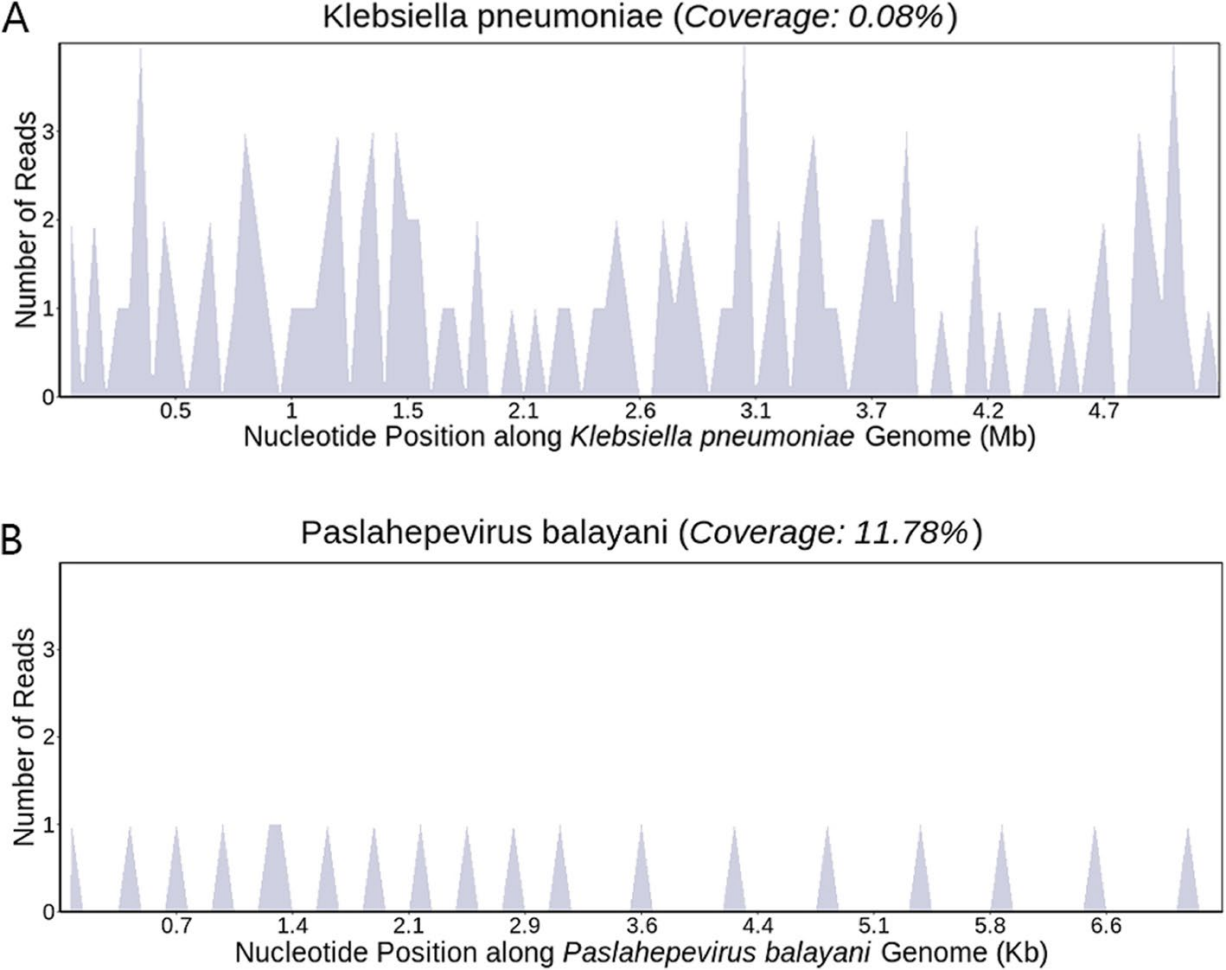
The genome coverage map. **A** The genome coverage map of *Klebsiella pneumoniae*. **B** The genome coverage map of *Paslahepevirus balayani*

Considering the seriousness of central nervous system infection, traditional detection and mNGS were applied for blood and CSF samples. On April 9, mNGS analysis of both CSF and peripheral blood unveiled an infection involving *K. pneumoniae* and HEV, as illustrated in Fig. 2. Quantitative real-time PCR assessment of HEV in peripheral blood (HEV-RNA) indicated a viral load of 1.8E + 04 copies/ml, and in feces (HEV-RNA), a viral load of 8.4E + 03 copies/ml. On April 19, the peripheral blood culture results from the previous hospital were obtained and confirmed *K. pneumoniae* infection (which was only resistant to ampicillin and was sensitive to amoxicillin, clavulanate, tobramycin, gentamicin, amikacin, chloramphenicol, cefazolin, cefathiamidine, cefuroxime, cetohexazine, piperacillin tazobactam, aztreonam, meropenem, ciprofloxacin, levofloxacin, moxifloxacin, tetracycline, trimethoprim and sulfamethoxazole.).

On the day of admission, the patient received treatment with isoglycoside magnesium oxalate injection (150 mg once daily), polyene phosphatidylcholine injection (697.5 mg once daily) for liver protection, and was administered mannitol (150 ml every 6 h) and glycerol fructose (150 ml every 12 h) to reduce intracranial pressure. Additionally, the patient received linezolid glucose injection (0.6 g every 12 h) and meropenem injection (2 g every 8 h) to empiric anti-infection therapy. Dexamethasone injection was given at a dose of 5 mg once daily for 7 days for its anti-inflammatory effects. The patient also took the oral powder (0.5 g twice daily) containing *Saccharomyces Boulardii* CNCM I-745, a fungal probiotic, to regulate intestinal flora and alleviated diarrhea symptom. Based on the result of CSF mNGS on April 9, linezolid was stopped. On April 19, the anti-infection medication was changed to ceftazidime injection (2 g every 8 h) until the patient was discharged on April 26.

Following treatment, the patient's fever subsided, and the headache improved. Re-examinations indicated a reduction in inflammation markers and improved liver function. A CSF test performed on April 18 showed clear and colorless fluid with a pressure of 95 mmH_2_O. On April 19, PCR testing did not detect HEV-RNA in the patient's serum and blood. All indicators were found to be within the normal range, as depicted in Table 2.

**Table 2:** Laboratory indicators during hospitalization

	4/5	4/8	4/9	4/10	4/11	4/12	4/13	4/17	4/22
WBC(E + 09/L)	14	10.03	10.01	6.51	8.47	7.51	9.07	7.71	5.39
N(E +09/L))	12.3	8.33	7.51	5.59	6.4	4.79	5.35	5.1	2.74
CRP(mg/L)	37.5	100.69	74.6	39.47	19.39	12.55	9.84	6.52	1.37
2003PCT(ng/ml)	4.73	2.64	2.09	1.51	0.72	0.43			
IL-6(pg/mL)		114.9				16			
SAA(mg/L)		378.7	298.4	104	18.3	5.4	9.8	8.2	2.3
ALT(U/L)	1780	378.7	267		206.6		168.5	88	40
AST(U/L)	596	423.9	32		59		45	29	24
ALP(U/L)	327	53	216		202		209	220	201
GGT(U/L)	292	235	201		207		214	192	145
LDH(U/L)	375	241	158		146		165	176	144
TBIL(μmol/L)	19.8	201	15.5		10.3		7.5	9.3	10.9
ALB(g/L)	40.5	17.7	25.7		26.1		29	32.4	36.1

## Discussion and conclusions

We present an unusual case involving simultaneous infections with hepatitis E virus and *Klebsiella pneumoniae* in both the central nervous system and bloodstream. The presence of positive Anti-HEV IgM test results and the detection of nucleic acid in peripheral blood, feces, and cerebrospinal fluid affirmed the diagnosis of acute hepatitis. Concurrently, there were significantly elevated levels of inflammatory markers upon admission. The cerebrospinal fluid displayed turbidity, and tests indicated acute suppurative meningitis. mNGS confirmed the presence of both HEV and *K. pneumoniae* in both cerebrospinal fluid and blood samples, establishing infections in both the central nervous system and bloodstream.

The transmission of Hepatitis E virus typically occurs via the gastrointestinal tract. Prior to the onset of symptoms, the patient attended a large banquet, which could potentially be the source of infection. Hepatitis E virus frequently manifests as acute liver inflammation accompanied by gastrointestinal symptoms such as fever, nausea, abdominal distension, and diarrhea. These symptoms correspond to the initial ones observed in the patient.

Upon entering the human body through the gastrointestinal tract, recent research suggests that HEV initially replicates in intestinal cells. Subsequently, it infects hepatocytes, and then spreads into the bloodstream, where it targets extrahepatic cells, including nerve cells. Upon release from the apical side, HEV is excreted through feces and urine [14]. By the 8th day of the disease's progression, varying levels of HEV were detected in the feces and peripheral blood. However, on the 18th day, no HEV was detectable, aligning with findings from previous studies [15].

Although HEV is primarily regarded as a virus that targets the liver, it can also lead to infections in the central nervous system by crossing the blood–brain barrier. In this instance, HEV was additionally identified in the cerebrospinal fluid, signifying an infection within the central nervous system.

*Klebsiella pneumoniae* holds significance within the *Klebsiella* genus of the *Enterobacteriaceae* family, commonly found in both the upper respiratory tract and the intestinal tract of humans. This bacterium assumes the role of an opportunistic pathogen, often linked to hospital-acquired infections [16, 17]. It primarily impacts individuals in hospital settings or those with compromised immune systems, particularly patients with pre-existing conditions like liver disease and diabetes [18].

The patient's counts of CD3 + total T cells, CD4 + helper T cells, and CD19 + B lymphocytes have all seen notable reductions, suggesting a deficiency in cellular immunity. This deficiency could heighten the risk of co-infection involving the HEV and *K. pneumoniae*. While the sequence of infections remains uncertain, it is posited that the patient might have initially contracted the Hepatitis E virus. This, in turn, could have caused diarrhea and significant liver dysfunction, thus permitting *K. pneumoniae* to breach the intestinal mucosal barrier, enter the bloodstream, and subsequently traverse the blood–brain barrier, resulting in an infection within the central nervous system.

Infections of the nervous system are potential life-threatening and prompt recognition and treatment of a central nervous system(CNS) infection is crucial for patient survival, as these infections have a high morbidity and mortality. Because of notable advantages in terms of timeliness and sensitivity, mNGS has a great diagnostic value in CNS infections and had an overall superior detection rate to culture [19–21]. In this case, mNGS successfully detected *K. pneumoniae* and HEV in CSF, while the culture results were negative. This case suggests that mNGS may be a useful diagnostic tool for CNS infection.

We present an exceptional case involving the simultaneous infection of the Hepatitis E virus and *K. pneumoniae* detected within a patient's cerebrospinal fluid and Peripheral blood. This case underscores the advantages of mNGS in the context of intricate infections.

## Data Availability

The datasets used and/or analysed during the current study are available from the corresponding author on reasonable request.

## Abbreviations

mNGS: Metagenomic next-generation sequencing
HEV: Hepatitis E virus
GBS: Guillain − Barré syndrome
CSF: Cerebrospinal fluid
ALT: Alanine aminotransferase
MRI: Magnetic resonance imaging
WBC: White blood cell
CRP: C-reactive protein
PCT: Procalcitonin
IL: Interleukin
PCR: Polymerase chain reaction

## References

[1] Kamar N, Izopet J, Pavio N, Aggarwal R, Labrique A, Wedemeyer H, Dalton HR. Hepatitis E virus infection. Nat Rev Dis Primers. 2017;3:17086. 10.1038/nrdp.2017.86 29154369

[2] European Association for the Study of the Liver (2018). Electronic address eee, European Association for the study of the L: EASL clinical practice guidelines on Hepatitis E virus infection. J Hepatol.

[3] Dalton HR, Kamar N, van Eijk JJJ, McLean BN, Cintas P, Bendall RP, Jacobs BC (2015). Hepatitis E virus and neurological injury. Nat Reviews Neurol.

[4] Abravanel F, Nicot F, Lhomme S, Cazabat M, Drumel T, Velay A, Latour J, Belliere J, Cintas P, Kamar N (2021). Hepatitis E Virus Quasispecies in Cerebrospinal Fluid with Neurological Manifestations. Vaccines.

[5] Kamar N, Izopet J, Cintas P, Garrouste C, Uro-Coste E, Cointault O, Rostaing L (2010). Hepatitis E Virus‐Induced neurological symptoms in a kidney‐transplant patient with chronic Hepatitis. Am J Transplant.

[6] Rahmig J, Grey A, Berning M, Schaefer J, Lesser M, Reichmann H, Puetz V, Barlinn K, Siepmann T (2020). Disseminated inflammation of the central nervous system associated with acute hepatitis E: a case report. BMC Neurol.

[7] Salim OJ, Davidson A, Li K, Leach JP, Heath C. Brainstem encephalitis and acute polyneuropathy associated with hepatitis E Infection. BMJ Case Rep. 2017;2017:bcr2017220799. 10.1136/bcr-2017-220799 PMC5623255 28899886

[8] Zhou X, Huang F, Xu L, Lin Z, de Vrij FMS, Ayo-Martin AC, van der Kroeg M, Zhao M, Yin Y, Wang W (2017). Hepatitis E Virus infects neurons and brains. J Infect Dis.

[9] Fritz M, Berger B, Schemmerer M, Endres D, Wenzel JJ, Stich O, Panning M (2018). Pathological cerebrospinal fluid findings in patients with neuralgic amyotrophy and Acute Hepatitis E virus Infection. J Infect Dis.

[10] Tian J, Shi R, Liu T, She R, Wu Q, An J, Hao W, Soomro MH (2019). Brain Infection by Hepatitis E Virus probably via damage of the blood-brain barrier due to alterations of tight Junction proteins. Front Cell Infect Microbiol.

[11] Hu F, Yang F, Wang M, Xu X, Yang Y, Ding B, Zhu J, Hao M, Wu S, Qin X (2020). The colonization of Carbapenem-resistant Klebsiella pneumoniae: epidemiology, resistance mechanisms, and risk factors in patients admitted to Intensive Care Units in China. J Infect Dis.

[12] Podschun R, Ullmann U (1998). Klebsiella spp. as nosocomial pathogens: epidemiology, taxonomy, typing methods, and pathogenicity factors. Clin Microbiol Rev.

[13] Huang N, Jia H, Zhou B, Zhou C, Cao J, Liao W, Liu S, Wang L, Chen L, Chen L (2022). Hypervirulent carbapenem-resistant Klebsiella pneumoniae causing highly fatal meningitis in southeastern China. Frontiers in Public Health.

[14] Mallet V, Scarano Pereira J-P, Martinino A, Roque-Afonso A-M (2021). The rise of the hepatitis E virus. J Hepatol.

[15] Ripellino P, Pasi E, Melli G, Staedler C, Fraga M, Moradpour D, Sahli R, Aubert V, Martinetti G, Bihl F (2019). Neurologic Complications of acute hepatitis E virus Infection. Neurol Neuroimmunol Neuroinflamm.

[16] Luo K, Tang J, Qu Y, Yang X, Zhang L, Chen Z, Kuang L, Su M, Mu D (2021). Nosocomial Infection by Klebsiella pneumoniae among neonates: a molecular epidemiological study. J Hosp Infect.

[17] Gorrie CL, Mirčeta M, Wick RR, Judd LM, Lam MMC, Gomi R, Abbott IJ, Thomson NR, Strugnell RA, Pratt NF (2022). Genomic dissection of Klebsiella pneumoniae infections in hospital patients reveals insights into an opportunistic pathogen. Nature Communications.

[18] Wyres KL, Lam MMC, Holt KE (2020). Population genomics of Klebsiella pneumoniae. Nat Rev Microbiol.

[19] Zhang Y, Cui P, Zhang HC, Wu HL, Ye MZ, Zhu YM, Ai JW, Zhang WH (2020). Clinical application and evaluation of metagenomic next-generation sequencing in suspected adult central nervous system Infection. J Transl Med.

[20] Miller S, Naccache SN, Samayoa E, Messacar K, Arevalo S, Federman S, Stryke D, Pham E, Fung B, Bolosky WJ (2019). Laboratory validation of a clinical metagenomic sequencing assay for pathogen detection in cerebrospinal fluid. Genome Res.

[21] Zhang S, Wu G, Shi Y, Liu T, Xu L, Dai Y, Chang W, Ma X (2022). Understanding etiology of community-acquired central nervous system Infections using metagenomic next-generation sequencing. Front Cell Infect Microbiol.

